# The Snail signaling branch downstream of the TGF-β/Smad3 pathway mediates Rho activation and subsequent stress fiber formation

**DOI:** 10.1016/j.jbc.2023.105580

**Published:** 2023-12-21

**Authors:** Mitsuyoshi Motizuki, Takashi Yokoyama, Masao Saitoh, Keiji Miyazawa

**Affiliations:** 1 Department of Biochemistry, Graduate School of Medicine, University of Yamanashi, Yamanashi, Japan; 2 Center for Medical Education and Sciences, Graduate School of Medicine, University of Yamanashi, Yamanashi, Japan

**Keywords:** cell motility, EMT, Rho, Smad, Snail, TGF-β

## Abstract

Cancer cells acquire malignant phenotypes through an epithelial–mesenchymal transition, which is induced by environmental factors or extracellular signaling molecules, including transforming growth factor-β (TGF-β). Among epithelial–mesenchymal transition–associated cell responses, cell morphological changes and cell motility are closely associated with remodeling of the actin stress fibers. Here, we examined the TGF-β signaling pathways leading to these cell responses. Through knockdown experiments in A549 lung adenocarcinoma cells, we found that Smad3-mediated induction of Snail, but not that of Slug, is indispensable for morphological changes, stress fiber formation, and enhanced motility in cells stimulated with TGF-β. Ectopic expression of Snail in *SMAD3*-knockout cells rescued the defect in morphological changes and stress fiber formation by TGF-β, indicating that the role of Smad3 in these responses is to upregulate Snail expression. Mechanistically, Snail is required for TGF-β–induced upregulation of Wnt5b, which in turn activates RhoA and subsequent stress fiber formation in cooperation with phosphoinositide 3-kinase. However, ectopic expression of Snail in *SMAD3*-knockout cells failed to rescue the defect in cell motility enhancement by TGF-β, indicating that activation of the Smad3/Snail/Wnt5b axis is indispensable but not sufficient for enhancing cell motility; a Smad3-dependent but Snail-independent pathway to activate Rac1 is additionally required. Therefore, the Smad3-dependent pathway leading to enhanced cell motility has two branches: a Snail-dependent branch to activate RhoA and a Snail-independent branch to activate Rac1. Coordinated activation of these branches, together with activation of non-Smad signaling pathways, mediates enhanced cell motility induced by TGF-β.

Transforming growth factor-β (TGF-β) is a pleiotropic cytokine involved in the regulation of a wide variety of physiological processes ranging from embryogenesis to adult tissue homeostasis. Aberration of its activities often leads to pathogenic conditions, including cancer, fibrosis, and immune dysfunctions (1). Notably, TGF-β is known to have two opposite effects on tumor progression; it functions as a tumor suppressor through proliferation inhibition of nontransformed cells, and it promotes tumor progression of transformed cells by inducing an epithelial–mesenchymal transition (EMT), which results in destabilization of cell–cell adhesions, acquisition of spindle cell morphology, formation of stress fibers, and enhancement of cell motility (2). The EMT also endows cells with stemness and drug-resistant properties. These cell responses are primarily induced by the expression of EMT-associated transcription factors (EMT-TFs), which include Snail, Slug, ZEB1, ZEB2, and Twist (3). Signaling from TGF-β is known to induce expression of these EMT-TFs, and underlying mechanisms of the induction of EMT-TFs have been extensively explored (2). However, the mechanism by which these EMT-TFs induced by TGF-β regulate various EMT-associated cell properties remains poorly understood.

Signals from TGF-β are principally transmitted through the Smad signaling pathway characteristic to ligands of the TGF-β family. Upon ligand stimulation, Smad2 and Smad3, termed receptor-regulated Smads, are phosphorylated at their C termini by a TGF-β type-I receptor, then form a heterotrimeric complex with Smad4 and translocate into the nucleus. Activated Smad complexes subsequently regulate gene expression through genomic DNA binding in cooperation with other transcription factors and coactivators/corepressors (4). In addition, TGF-β transmits signals *via* non-Smad signaling pathways shared with other growth factors and cytokines (5). These pathways include the extracellular signal–regulated kinase1 and 2 (ERK1/2), c-Jun N-terminal kinase (JNK), p38 mitogen-activated protein kinase (MAPK), phosphoinositide 3-kinase (PI3K), and Src pathways. The ERK pathway is stimulated by an activated TGF-β type-I receptor through tyrosine phosphorylation of the adaptor protein Shc, enabling docking of the Grb2–Sos1 complex, which subsequently leads to downstream activation of the Ras/Raf/MEK/ERK pathway (6). The activation of JNK, p38 MAPK, and PI3K is mediated through TRAF4/6 (7, 8, 9). The Src pathway is initiated by TGF-β–stimulated tyrosine phosphorylation of the type-I receptor by the type-II receptor (10). Although EMT-associated cell responses (*i.e.*, cell morphological changes, stress fiber formation, and cell motility) are dependent on the Smad3 pathway (11, 12, 13), some non-Smad pathways are also indispensable; inhibition of p38 MAPK or PI3K with specific inhibitors abrogates TGF-β–induced actin reorganization (14, 15, 16, 17). Therefore, TGF-β–induced EMT is modulated *via* both the Smad and non-Smad signaling pathways.

The Rho family of small GTPases (Rho, Rac, and Cdc42) regulate the actin cytoskeleton and focal adhesion complexes, which are critical for morphogenesis, cell motility, and invasiveness (18). In two-dimensional cell migration, Rac1 and Cdc42 promote the formation of protrusions such as lamellipodia at the leading edge, whereas RhoA induces rear retraction and forward movement through the formation of contractile stress fibers at the trailing edge (19). TGF-β can rapidly activate RhoA and Rac1, contributing to EMT and the enhancement of cell motility (15, 16, 20). We have previously reported that Smad3-dependent downregulation of *ARHGAP24* as well as independent activation of PI3K contributes to the TGF-β–induced activation of Rac1 (11). Although we observed that the Smad3 pathway is also required for stress fiber formation (11), the process by which Smad3 mediates the activation of RhoA to induce stress fiber formation remains insufficiently understood.

In the present study, we elucidated the pathway that induces RhoA activation downstream of Smad3, using A549 lung adenocarcinoma cells. The role of Smad3 in TGF-β–induced cell morphological changes and stress fiber formation is to upregulate the expression of Snail, which in turn induces Wnt5b, along with the MEK/ERK pathway signaling. Wnt5b activates RhoA to induce stress fiber formation and cell morphological changes in cooperation with PI3K. However, knockdown of Snail did not affect E-cadherin downregulation by TGF-β even though the downregulation is also Smad3 dependent. Our present findings thus indicate that EMT-associated cell responses induced by TGF-β are likely to be regulated by distinct pathways that branch at Smad3.

## Results

### Snail is indispensable for stress fiber formation, cell morphological changes, and enhanced cell motility induced by TGF-β

**Figure 1:**
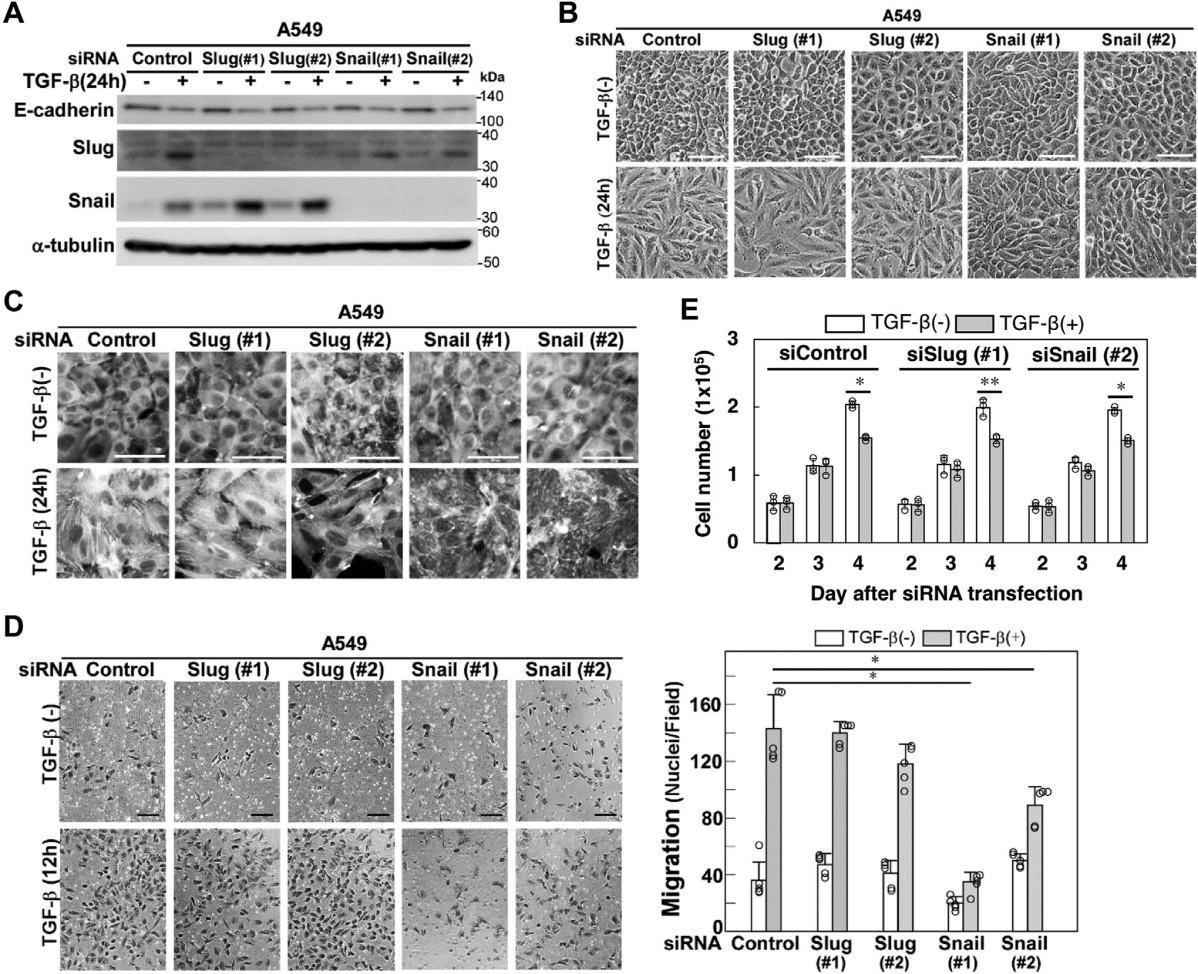
**Effects of Slug or Snail knockdown on cell responses induced by TGF-β**. *A–E*, A549 cells were transfected with two different siRNAs against Slug (#1 or #2), Snail (#1 or #2), or control siRNA. After 48 h, cells were stimulated with 1 ng/ml of TGF-β1 for the indicated time. *A*, expression of E-cadherin, Slug, or Snail was determined by immunoblotting; α-tubulin was used as a loading control. *B*, light microscopic images. *C*, formation of actin stress fibers. F-actin was stained using Rhodamine phalloidin. *D*, chamber migration assay. TGF-β stimulation for 12 h. Quantification is shown on the *right*. Error bars represent the SD (*n* = 5). The *p* values were determined by Dunnett’s multiple comparison test ∗*p* < 0.01. *E*, cell proliferation evaluated by cell number counting (triplicate determination). The *p* values were determined by Student's *t* test. ∗*p* < 0.05; ∗∗*p* < 0.01. TGF-β1 was added on day 2. *Scale bars*: 100 μm (*B–D*). One representative result from two independent experiments is shown (*B–E*). TGF-β, transforming growth factor-β.

Snail and Slug belong to the zinc-finger family transcription factors and are known to be involved in EMT (21). Here, we examined the roles of Snail and Slug in TGF-β–induced EMT using A549 lung adenocarcinoma cells. Snail or Slug was knocked down using siRNA duplexes, and the knockdown was verified by immunoblotting (Fig. 1*A*). As we previously reported, knockdown of Slug resulted in upregulation of Snail (22), whereas knockdown of Snail slightly repressed Slug expression. Under these conditions, various cellular responses induced by TGF-β were examined (Fig. 1, *B*–*E*). Knockdown of Snail attenuated TGF-β–induced cell morphological changes (Fig. 1*B*), stress fiber formation (Fig. 1*C*), and cell motility (Fig. 1*D*) but not E-cadherin downregulation (Fig. 1*A*) or cytostasis (Fig. 1*E*). A similar effect of Snail knockdown on stress fiber formation was observed in PANC-1 pancreatic cancer cells (Fig. S1*A*). By contrast, knockdown of Slug did not affect all of these cell responses. Therefore, we subsequently focused on the role of Snail in cell responses induced by TGF-β.

### Snail expression is not sufficient for cell morphological changes, stress fiber formation, or cell motility induced by TGF-β

**Figure 2:**
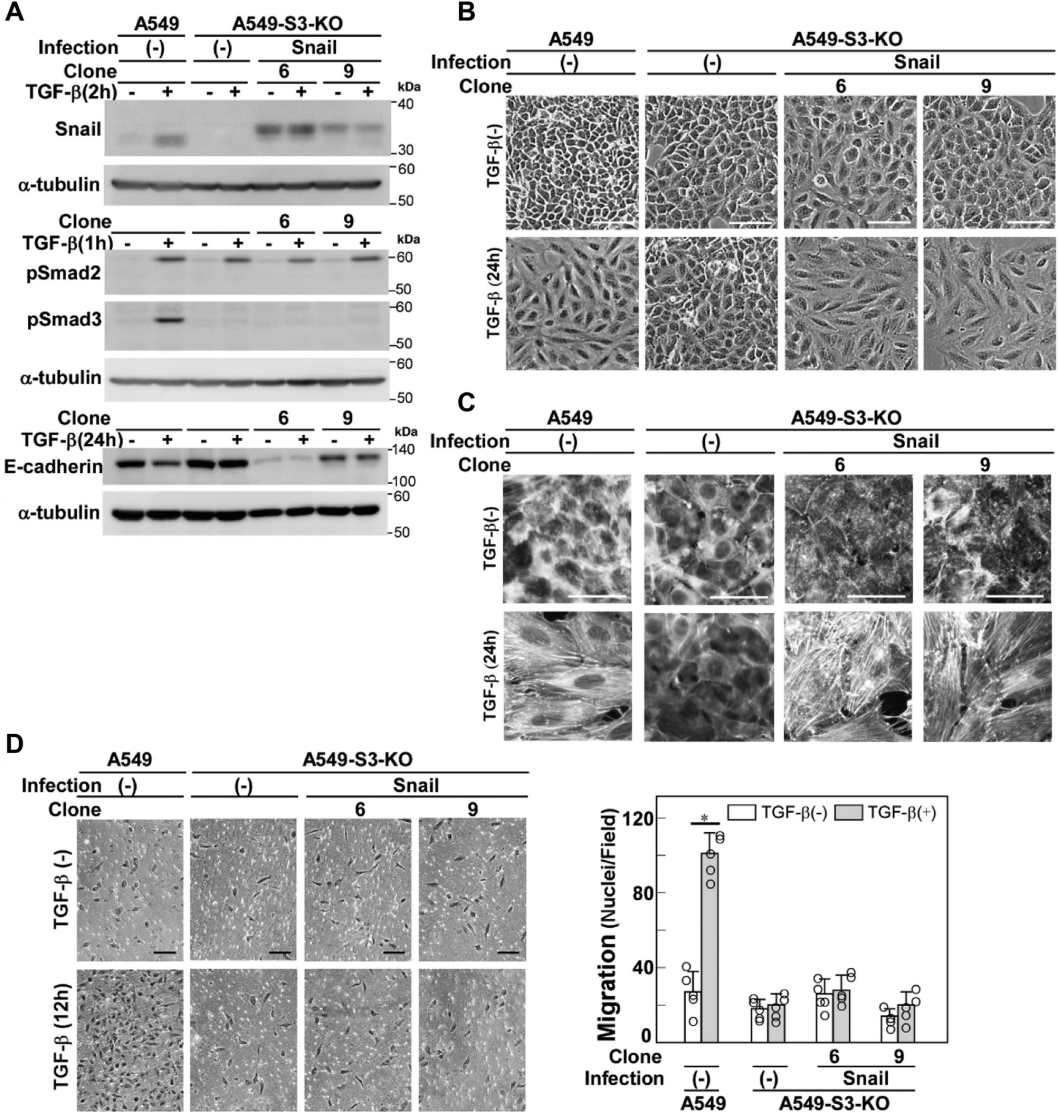
**Exogenous Snail rescues the defect of TGF-β–induced cell morphological changes and stress fiber formation in *SMAD3*-knockout A549 cells**. *SMAD3-*knockout A549 cells (A549-S3-KO) were infected with lentivirus carrying cDNA encoding HA-tagged Snail encoding cDNA. *A–D*, A549 cells, A549-S3-KO cells, or those expressing Snail were incubated in either the presence or absence of 1 ng/ml of TGF-β1 for the indicated time. *A*, immunoblot analysis of expression levels of Snail, phospho-Smad2/3, and E-cadherin; α-tubulin was used as a loading control. *B*, light microscopic images. *C*, formation of actin stress fibers. F-actin was stained using Rhodamine phalloidin. *D*, chamber migration assay. TGF-β stimulation for 12 h. Quantification is shown on the *right*. Error bars represent the SD (*n* = 5). The *p* values were determined by Student's *t* test. ∗*p* < 0.01. *Scale bars*: 100 μm (*B–D*). One representative result from two independent experiments is shown (*B–D*). TGF-β, transforming growth factor-β.

EMT-associated cell responses affected by Snail knockdown in A549 cells (Fig. 1, cell morphological changes, stress fiber formation, and cell motility) are all dependent on Smad3 (11). Therefore, we examined how Smad3 modulates the Snail pathway to induce stress fiber formation, cell morphological changes, and cell motility enhancement. We first examined the induction of Snail protein in *SMAD3*-knockout A549 (A549-S3-KO) cells. In a previous report, the induction of Snail by TGF-β was found to be Smad3-dependent in renal tubular epithelial cells (23); we found that the induction was also Smad3-dependent in the A549 cells (Fig. 2*A*). We introduced exogenous Snail by lentiviral infection in A549-S3-KO cells and examined whether the Snail expression rescues TGF-β–induced cell responses. We selected two clones for further analyses: one clone expressing a higher level and another expressing a lower level of Snail protein compared with that of endogenous Snail induced by TGF-β (Fig. 2*A*). Expression of exogenous Snail did not induce spindle cell morphologies in A549-S3-KO cells; however, additional TGF-β stimulation induced robust cell morphological changes in these cells (Fig. 2*B*). Similarly, exogenous Snail expression failed to induce stress fiber formation, which was effectively facilitated by TGF-β stimulation (Fig. 2*C*). Therefore, the defect in TGF-β–induced cell morphological changes and stress fiber formation introduced by *SMAD3*-knockout were rescued by exogenous Snail expression, indicating that the role of Smad3 in these responses is to upregulate Snail protein expression. However, expression of Snail itself was not sufficient to induce cell morphological changes or stress fiber formation; additional TGF-β signaling other than the Smad3 pathway appears to be involved. By contrast, exogenous Snail failed to rescue the defect in enhancement of cell motility by TGF-β in A549-S3-KO cells (Fig. 2*D*). Some other branch(es) of Smad3-dependent signaling pathways are likely required for the cell motility enhancement. Collectively, these results show that Snail expression is indispensable but not sufficient for cell morphological changes, stress fiber formation, or cell motility enhancement induced by TGF-β.

As shown in Figure 1*A*, TGF-β–induced downregulation of E-cadherin expression was not attenuated by knockdown of Snail, indicating that induction of endogenous Snail is not involved in the E-cadherin downregulation. However, expression of exogenous Snail alone in A549-S3-KO cells resulted in substantial downregulation of E-cadherin expression (Fig. 2*A*). Therefore, the level or duration of endogenous Snail expression induced by TGF-β in A549 cells might not be sufficient for downregulating E-cadherin expression.

### Signaling *via* the PI3K pathway is additionally required for cell morphological changes and stress fiber formation induced by TGF-β

**Figure 3:**
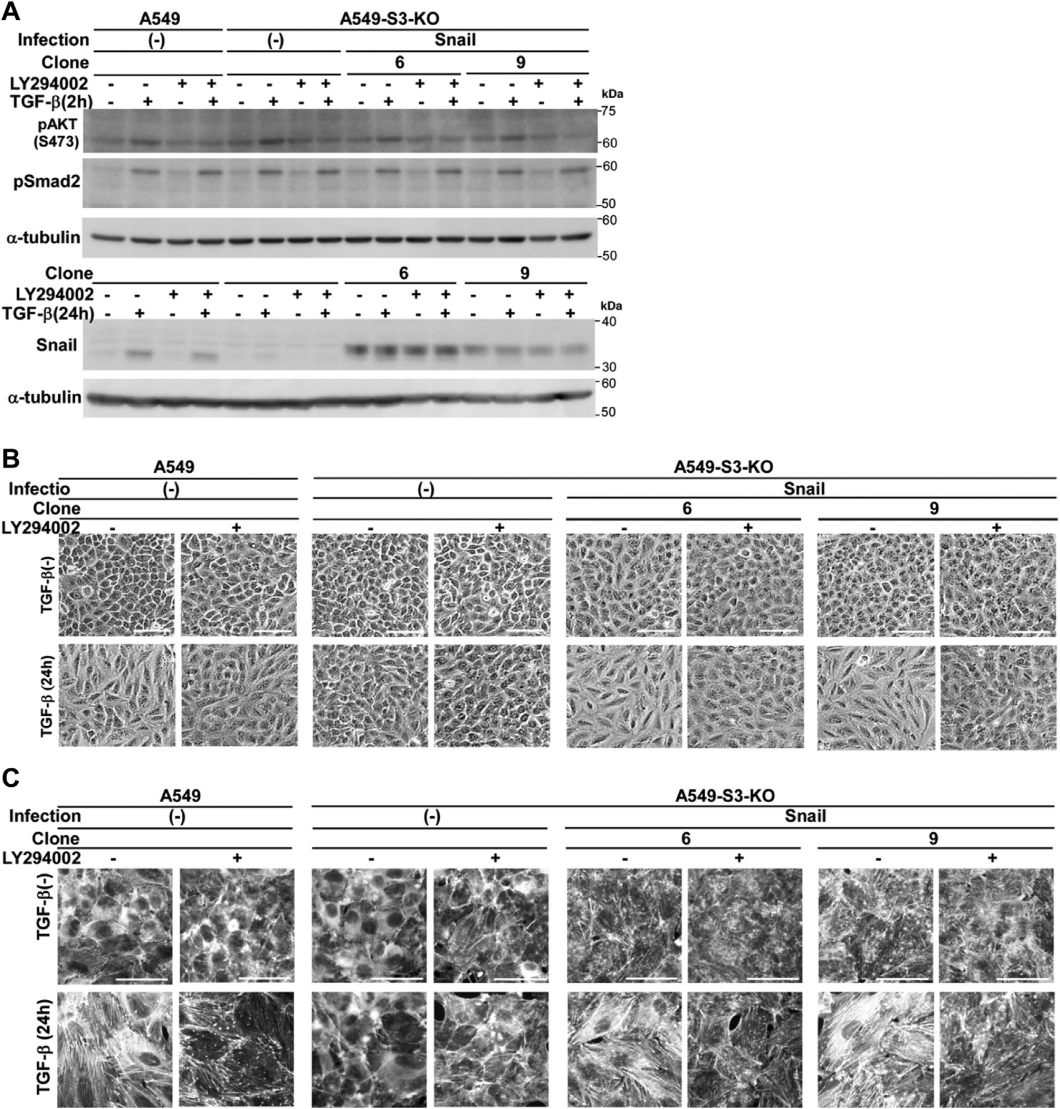
**Both PI3K and Snail are required for TGF-β–induced cell morphological changes and stress fiber formation.***A–C*, A549 cells, A549-S3-KO cells, or those expressing Snail were pretreated with either a PI3K inhibitor LY294002 (10 μM) or 0.1% dimethyl sulfoxide for 1 h and stimulated with 1 ng/ml of TGF-β1 for the indicated time. *A*, TGF-β–induced phosphorylation of Akt (S473). Cell lysates were analyzed by immunoblotting using anti–phospho-Akt, phospho-Smad2, or Snail antibodies; α-tubulin was used as a loading control. *B*, light microscopic images. *C*, formation of actin stress fibers. F-actin was stained using Rhodamine phalloidin. *Scale bars*: 100 μm (*B* and *C*). One representative result from two independent experiments is shown (*A–C*). PI3K, phosphoinositide 3-kinase; TGF-β, transforming growth factor-β.

Bakin *et al*. reported that stress fiber formation and cell motility induced by TGF-β are PI3K-dependent in NMuMG cells (14). Therefore, the PI3K pathway might be a good candidate for the signaling pathway required for cell morphological changes and stress fiber formation, in addition to the induction of Snail. We therefore examined the effects of LY294002, a widely used PI3K inhibitor. LY294002 did not inhibit the induction of Snail by TGF-β, nor did it affect the expression levels of exogenous Snail; however, it blocked phosphorylation of Akt, a downstream effector of the PI3K pathway (Fig. 3*A*). As anticipated, LY294002 inhibited TGF-β–induced cell morphological changes and stress fiber formation in parental A549 cells as well as in A549-S3-KO cells expressing exogenous Snail protein (Fig. 3, *B* and *C*). Therefore, the PI3K signaling likely cooperates with Snail in inducing cell morphological changes and stress fiber formation.

### The Snail and PI3K pathways cooperate to activate RhoA but not Rac1

**Figure 4:**
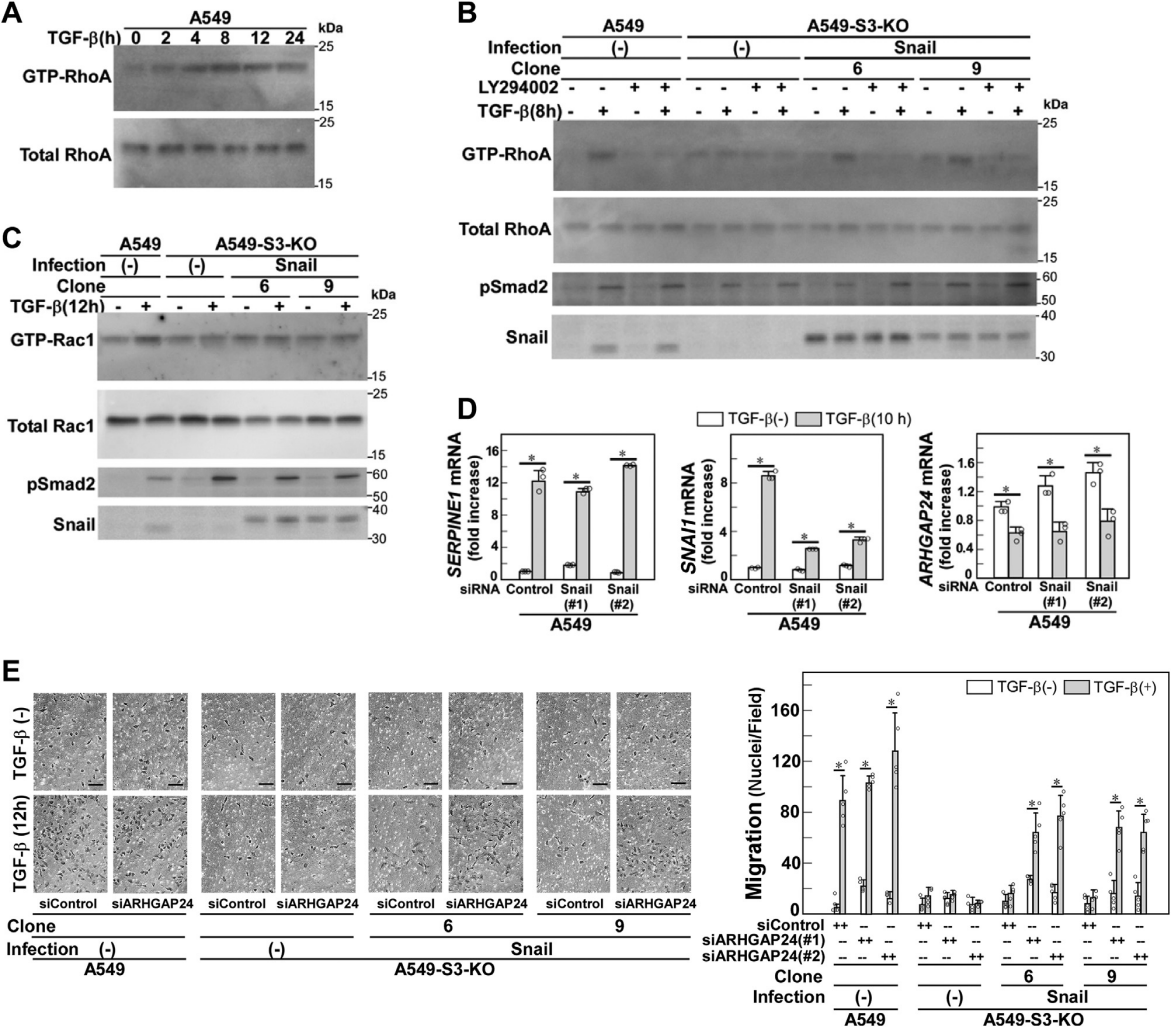
**The Snail pathway downstream of TGF-β is involved in RhoA activation.***A–E*, A549 cells, A549-S3-KO cells, or those expressing exogenous Snail were incubated in either the presence or absence of 1 ng/ml of TGF-β1 for the indicated time. The amount of active, GTP-loaded Rac1 or RhoA was determined by GST pull-down followed by immunoblotting. *A*, a time course showing RhoA activation after TGF-β stimulation. *B*, involvement of Snail in TGF-β–induced activation of RhoA. Cells were pretreated with either a PI3K inhibitor LY294002 (10 μM) or 0.1% DMSO for 1 h and then stimulated with TGF-β1 for 8 h. *C*, irrelation of Snail in TGF-β–induced activation of Rac1. *D*, Snail knockdown does not affect *ARHGAP24* downregulation induced by TGF-β. Cells were stimulated with TGF-β1 for 10 h on collagen-coated plates, harvested, and subjected to quantitative real-time PCR. *E*, effect of *ARHGAP24* knockdown on TGF-β–induced motility in A549-S3-KO cells expressing exogenous Snail. Cells were treated with control siRNA (siControl) or two different siRNAs against *ARHGAP24* (#1 or #2) for 24 h and harvested for chamber migration assay. TGF-β stimulation for 12 h. Quantification is shown on the *right*. *Scale bars*: 100 μm (*E*). Error bars represent the SD (*n* = 3 for *D* and *n* = 5 for *E*). The *p* values were determined by Student’s *t* test. ∗*p* < 0.01. One representative result from two independent experiments is shown (*A–E*). DMSO, dimethyl sulfoxide; TGF-β, transforming growth factor-β.

The roles of Rho activation in cell morphological changes and stress fiber formation induced by TGF-β have been extensively explored (16, 20, 24, 25, 26). We examined the possible involvement of the Snail and PI3K signaling pathways in Rho activation. In A549 cells, RhoA activation by TGF-β was detected from 2 to 24 h, peaking at 8 h after stimulation (Fig. 4*A*). Therefore, we examined the RhoA activation status after TGF-β stimulation (8 h) in A549 parental cells as well as in A549-S3-KO cells with or without exogenous Snail expression (Fig. 4*B*). In A549 parental cells, LY294002 inhibited RhoA activation without attenuating Smad phosphorylation. In A549-S3-KO cells, RhoA activation by TGF-β was abrogated but was rescued by exogenous Snail expression, which was again attenuated by LY294002 without affecting the expression level of either Snail or phospho-Smad2. Therefore, RhoA activation by TGF-β requires both the Snail and PI3K signaling pathways.

We previously examined the signaling pathways leading to activation of Rac1, another small G protein involved in cell motility (11). Activation of Rac1 is dependent on the Smad3 and PI3K pathways, the former of which dictates downregulation of *ARHGAP24*, a GTPase-activating protein that negatively regulates Rac1. Exogenous Snail expression failed to rescue defective Rac1 activation in A549-S3-KO cells (Fig. 4*C*), and downregulation of *ARHGAP24* by TGF-β was Snail independent (Fig. 4*D*). Interestingly, cell motility enhancement by TGF-β was recovered when *ARHGAP24* was knocked down in A549-S3-KO cells expressing exogenous Snail (Fig. 4*E*). These findings indicate that two branches of the Smad3 pathway—one leading to induction of Snail and the other leading to downregulation of *ARHGAP24—*cooperate with the PI3K pathway to enhance cell motility.

### Wnt5b links induction of Snail and activation of RhoA

**Figure 5:**
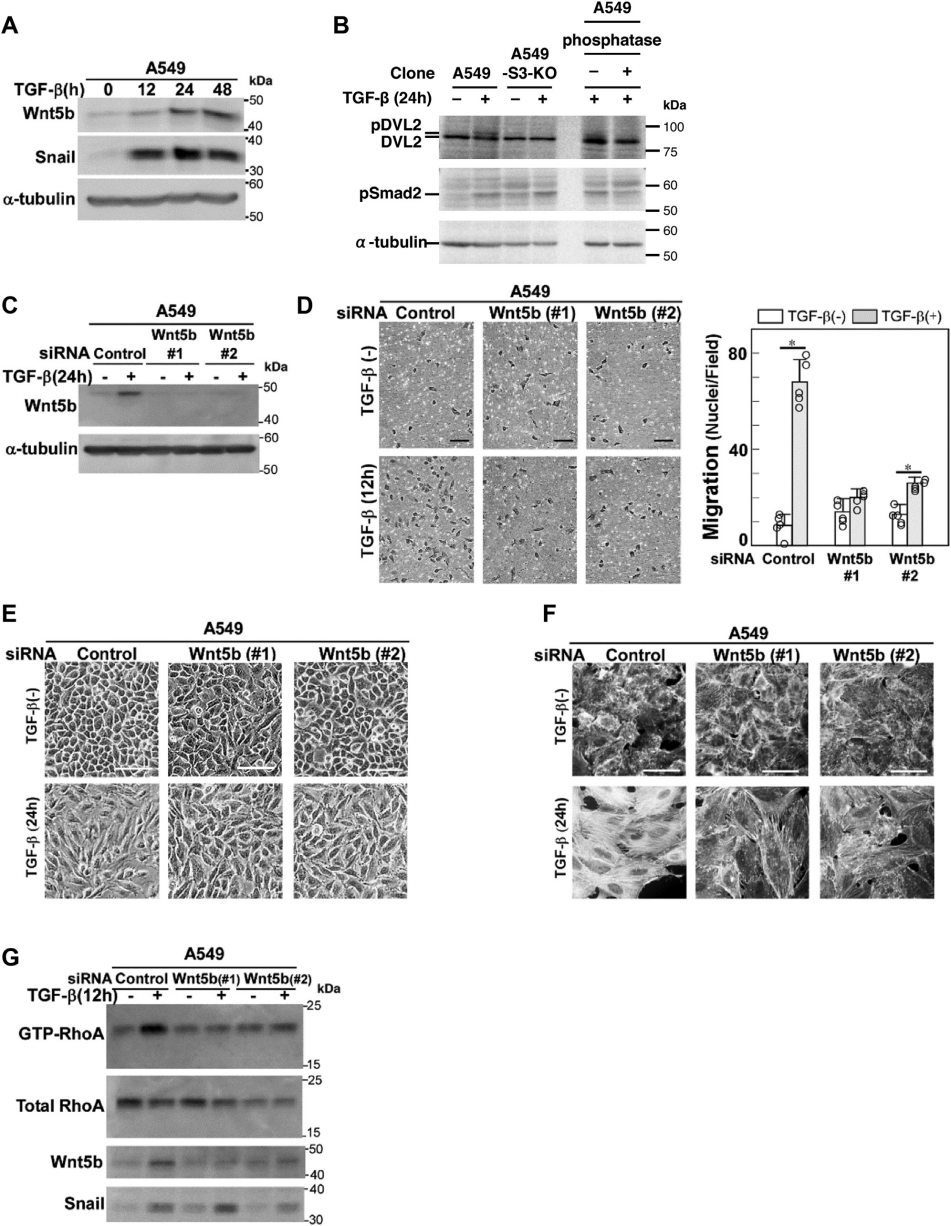
**Wnt5b is indispensable for RhoA activation induced by TGF-β.***A*, time course of Wnt5b expression after TGF-β stimulation (1 ng/ml of TGF-β1). Wnt5b and Snail were detected by immunoblotting. *B*, A549 cells or A549-S3-KO cells were stimulated with 1 ng/ml of TGF-β1 for 24 h. Cell lysates were analyzed by immunoblotting using anti-DVL2 or phospho-Smad2 antibodies; α-tubulin was used as a loading control. The phosphorylation of mobility-shifted DVL2 was confirmed by alkaline phosphatase treatment. *C–G*, A549 cells were transfected with two different siRNAs against Wnt5b (#1 or #2) or control siRNA. After 24 h, cells were stimulated with 1 ng/ml of TGF-β1 for the indicated time. *C*, knockdown of Wnt5b was verified by immunoblotting; α-tubulin was used as a loading control. *D*, chamber migration assay. TGF-β stimulation for 12 h. Quantification is shown on the *right*. Error bars represent the SD (*n* = 5). The *p* values were determined by Student’s *t* test. ∗*p* < 0.01. *E*, light microscopic images. *F*, formation of actin stress fibers. F-actin was stained using Rhodamine phalloidin. *G*, TGF-β–induced activation of RhoA was inhibited by knockdown of Wnt5b. RhoA and Wnt5b were detected by immunoblotting. *Scale bars*: 100 μm (*D–F*). One representative result from two independent experiments is shown (*A–G*). TGF-β, transforming growth factor-β.

In Wingless/integrase 1 (WNT) signaling, the binding of WNT ligands to frizzled receptors induces an intracellular signaling cascade that involves cytoskeletal rearrangements and other events (27). WNT proteins constitute a family of secreted glycoproteins, consisting of 19 ligands known in humans. Of these WNT ligands, *WNT5B* expression is significantly induced by TGF-β in A549 cells (GSE109296). Intriguingly, Wnt5b has been reported to enhance cell motility by activating RhoA (28). Therefore, we examined the role of Wnt5b in the TGF-β–induced stress fiber formation. Wnt5b protein expression was induced by TGF-β and almost plateaued 24 h after stimulation (Fig. 5*A*). Consistently, DVL2 (Dishevelled Segment Polarity Protein 2), a downstream effector of Wnt5b, was phosphorylated in a Smad3-dependent manner at 24 h after TGF-β stimulation (Fig. 5*B*). We then knocked down endogenous Wnt5b using siRNA duplexes, which was verified by immunoblotting (Fig. 5*C*). Wnt5b knockdown attenuated TGF-β–induced cell motility (Fig. 5*D*). Even after TGF-β stimulation, many of the cells with Wnt5b knockdown exhibited a cobblestone-like shape rather than a spindle shape and had fewer stress fibers (Fig. 5, *E* and *F*). In addition, knockdown of Wnt5b attenuated activation of RhoA induced by TGF-β (Fig. 5*G*). These Wnt5b-dependent responses were also observed in PANC-1 cells (Fig. S1, *B* and *C*). Although Wnt5a has been reported to be induced by TGF-β in airway smooth muscle cells and mammary gland cells (29, 30), *WNT5A* was not induced by TGF-β in A549 cells in the present study (Fig. S2*A*). These results suggest that Wnt5b is a key Wnt ligand that promotes TGF-β–induced rearrangement of the actin cytoskeleton in A549 cells.

**Figure 6:**
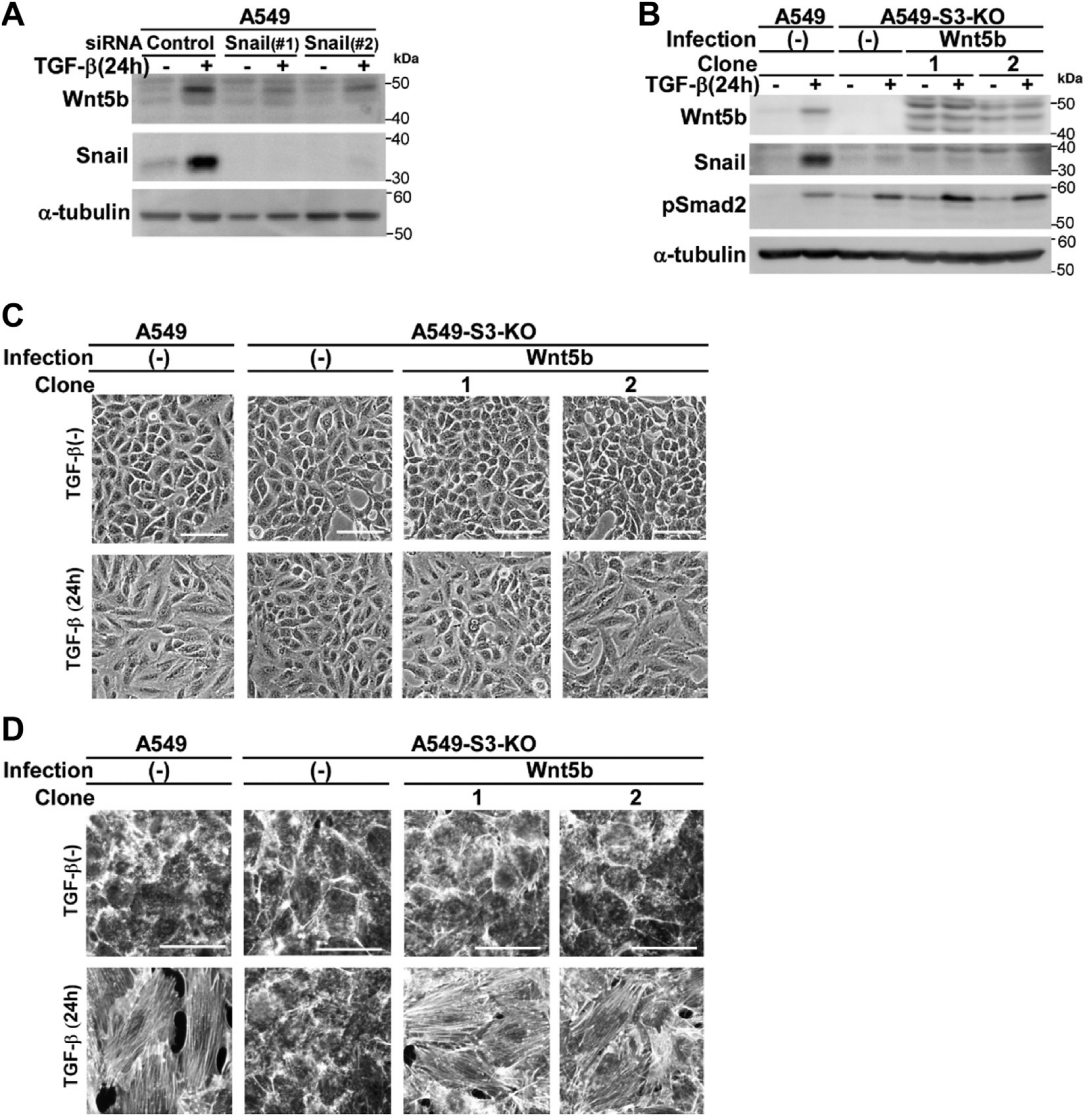
**Exogenous Wnt5b rescues the defect of TGF-β–induced cell morphological changes and stress fiber formation in *SMAD3*-knockout A549 cells**. *A*, TGF-β–induced expression of Wnt5b was inhibited by knockdown of Snail. A549 cells were transfected with two different siRNAs against Snail (#1 or #2) or control siRNA. After 24 h, cells were stimulated with 1 ng/ml of TGF-β1 for 24 h. Wnt5b and Snail were detected by immunoblotting. *B–D*, A549-S3-KO cells were infected with lentivirus carrying cDNA encoding Flag-tagged Wnt5b encoding cDNA. A549 cells, A549-S3-KO cells, or those expressing exogenous Wnt5b were incubated in either the presence or absence of 1 ng/ml of TGF-β1 for 24 h. *B*, expression of Wnt5b, Snail, or phopho-Smad2 was detected by immunoblotting; α-tubulin was used as a loading control. *C*, light microscopic images. *D*, formation of actin stress fibers. F-actin was stained using Rhodamine phalloidin. *Scale bars*: 100 μm (*C* and *D*). One representative result from two independent experiments is shown (*A–D*). TGF-β, transforming growth factor-β.

Importantly, knockdown of Snail inhibited induction of Wnt5b by TGF-β in A549 (Fig. 6*A*) and PANC-1 cells (Fig. S1*D*), suggesting that Wnt5b is a downstream effector of Snail. We then introduced exogenous *WNT5B* by lentiviral infection of A549-S3-KO cells and examined whether *WNT5B* expression rescues TGF-β–induced cell responses. Two clones expressing Wnt5b protein at levels comparable to that induced by TGF-β were selected for further analyses (Fig. 6*B*). Exogenous Wnt5b expression itself did not induce cell morphological changes or stress fiber formation but rescued the defect in these responses in A549-S3-KO cells stimulated with TGF-β (Fig. 6, *C* and *D*). These results indicate that the role of Snail in RhoA activation by TGF-β, as well as the accompanying cell morphological changes and stress fiber formation, is attributable to the induction of Wnt5b expression.

### Involvement of non-Smad signaling pathways in TGF-β–induced cell morphological changes and stress fiber formation

**Figure 7:**
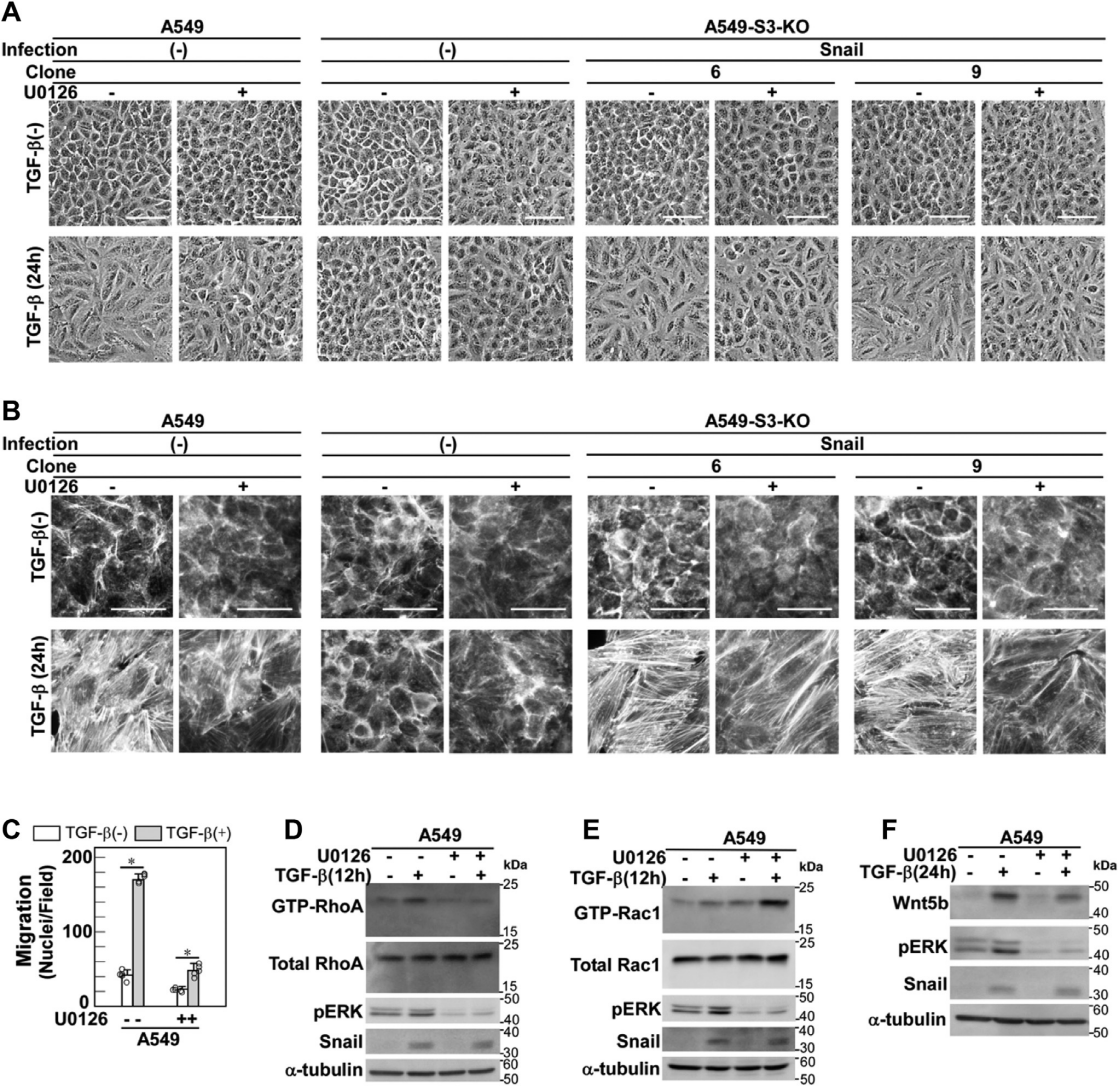
**A MEK inhibitor U0126 attenuates TGF-β–induced cell morphological changes, stress fiber formation, and cell motility.***A–F*, A549 cells, A549-S3-KO cells, or those expressing Snail were pretreated with either a MEK inhibitor U0126 (10 μM) or 0.1% DMSO for 1 h and stimulated with 1 ng/ml of TGF-β1 for the indicated time. *A*, light microscopic images. *B*, formation of actin stress fibers. F-actin was stained using Rhodamine phalloidin. *C*, chamber migration assay. TGF-β stimulation for 12 h. Error bars represent the SD (*n* = 5). The *p* values were determined by Student’s *t* test. ∗*p* < 0.01. *D*, TGF-β–induced activation of RhoA was inhibited by U0126. *E*, TGF-β–induced activation of Rac1 was not inhibited by U0126. *F*, TGF-β–induced expression of Wnt5b was inhibited by U0126. Wnt5b was detected by immunoblotting. *Scale bars*: 100 μm (*A* and *B*). One representative result from two independent experiments is shown (*A–F*). DMSO, dimethyl sulfoxide; TGF-β, transforming growth factor-β.

Because the role of Smad3 in cell morphological changes, stress fiber formation, and cell motility induced by TGF-β has been elucidated, we further examined the possible involvement of non-Smad signaling pathways other than the PI3K pathway (*i.e.*, the ERK, p38 MAPK, JNK, and c-Src pathways) (Figs. 7 and S3).

U0126, a MEK inhibitor, attenuated the cell morphological changes, stress fiber formation, and cell motility induced by TGF-β in A549 parental cells (Fig. 7, *A* and *B*, left panels, and *C*). Consistent with these findings, U0126 suppressed RhoA activation but not Rac1 activation (Fig. 7, *D* and *E*). Notably, the basal level of GTP-Rac1 was higher in U0126-treated cells than in control cells (Fig. 7*E*). RhoA and Rac1 are known to be antagonistic to each other (31). Therefore, inhibition of RhoA activation by U0126 was likely to result in enhancement of Rac1 activation. We also examined the effects of U0126 on cell morphological changes and stress fiber formation in A549-S3-KO cells expressing exogenous Snail. U0126 attenuated these cell responses (Fig. 7, *A* and *B*, right panels), indicating that the MEK/ERK pathway plays a role downstream of Snail. Consequently, we found that U0126 attenuates the induction of Wnt5b protein (Fig. 7*F*) and mRNA expression (Fig. S2), both of which are events downstream of Snail. These results suggest that activation of the MEK/ERK pathway is involved in TGF-β/Snail–mediated induction of Wnt5b, leading to activation of the Rho pathway and subsequent cell morphological changes, stress fiber formation, and cell motility.

We further examined the effects of inhibitors of p38 MAPK, JNK, or Src on TGF-β–induced morphological changes and stress fiber formation. The activation of p38, JNK, or Src by TGF-β was confirmed by immunoblot analysis (Fig. S3*A*). Consistent with the results of previous studies involving NMuMG cells (15, 16, 17), SB2032580, a p38 MAPK inhibitor, markedly inhibited TGF-β–induced morphological changes and stress fiber formation, whereas SP600125, a JNK inhibitor, failed to do so (Fig. S3, *B* and *C*). Recently, Src kinase activity was shown to be required for actin filament formation by TGF-β in mouse embryonic fibroblasts (10). Dasatinib, which inhibits Src kinase, affected cell morphology to some extent, even in the unstimulated condition. In addition, the morphologies of Dasatinib-treated cells after TGF-β stimulation differed substantially from those of Dasatinib-untreated cells (Fig. S3, *B* and *C*). Therefore, evaluating its effects in A549 cells is difficult. Collectively, the results show that the Smad3-dependent Snail pathway and non-Smad signaling pathways activated by TGF-β cooperate to induce cell morphological changes and stress fiber formation.

## Discussion

**Figure 8:**
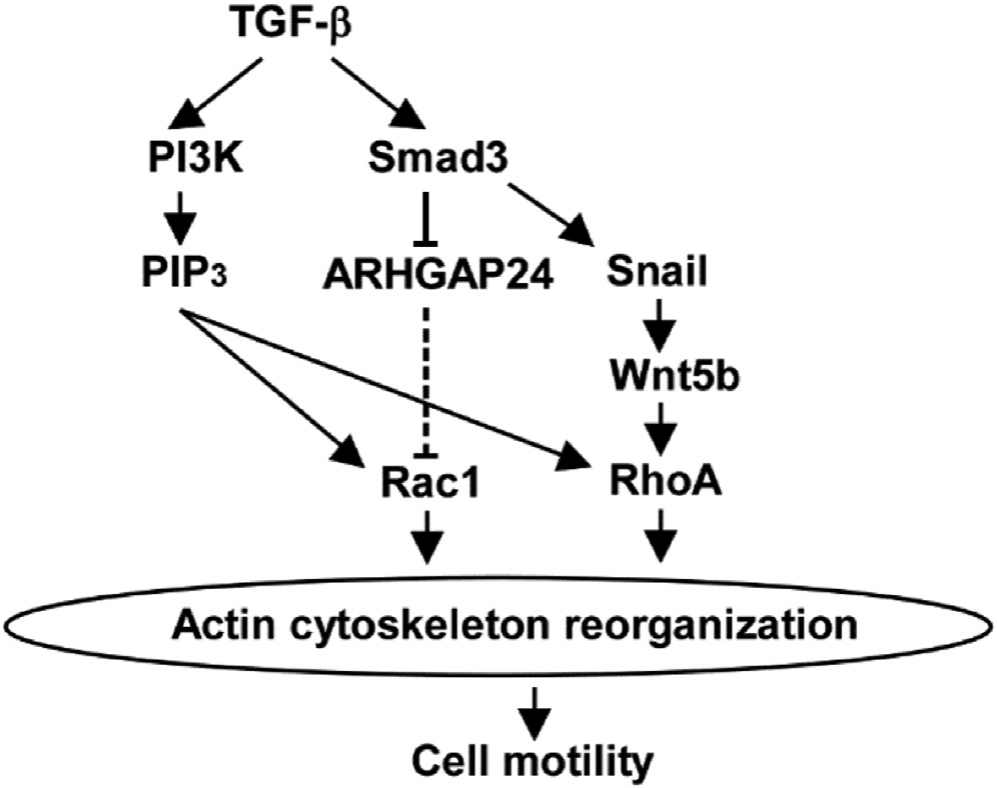
**Signaling pathways leading to actin cytoskeleton reorganization by TGF-β**. PI3K, phosphoinositide 3-kinase; PIP_3_, phosphatidylinositol-3,4,5-trisphosphate; TGF-β, transforming growth factor-β.

Snail and Slug are well-known members of the core EMT-associated transcription factors (21). Their roles in the process of EMT have been extensively explored over the past 2 decades (32). However, many of the related works have focused on how Snail or Slug is induced by extracellular stimuli or how ectopically expressed Snail or Slug induces EMT. The mechanism by which endogenous Snail or Slug drives TGF-β–induced EMT remains poorly understood. We have previously reported that cell motility induced by TGF-β is mediated through, at least in part, a pathway distinct from those mediating other EMT-associated responses (11, 33, 34, 35). In the present study, we further examined branches of the TGF-β signaling pathway leading to EMT-associated responses. We discovered that TGF-β promotes cell morphological changes and actin cytoskeleton reorganization through cooperation of two pathways: the Smad3/Snail/Wnt5b pathway and the PI3K pathway (Fig. 8).

Knockdown of Snail inhibited TGF-β–induced cell morphological changes, stress fiber formation, and motility enhancement, whereas knockdown of Slug had no effect on these EMT-associated cell responses in A549 cells (Fig. 1, *B*–*D*). Lentiviral expression of Snail rescued the defects in these cell responses in A549-S3-KO cells stimulated with TGF-β (Fig. 2*C*), whereas Slug expression did not (Fig. S4). These results suggest that Snail, but not Slug, is critical for TGF-β–induced EMT in A549 cells. However, Slug has been reported to play an important role in TGF-β–induced EMT in oral squamous cell carcinoma cells (36, 37, 38). Moreover, Slug is involved in the induction of Wnt5b in HSC-4 oral squamous cell carcinoma cells (38). Whether the induction of EMT-associated cell responses is dependent on Snail or Slug appears to vary among different cell types. The underlying mechanism for this selective dependency remains to be elucidated.

Snail is a zinc-finger transcription factor that promotes cell survival and metastasis through the induction of EMT (39). The migration and invasive capacities increase in cells overexpressing Snail and decrease when Snail is silenced (40, 41, 42, 43, 44). Although Snail expression exhibits a tight correlation with the invasive phenotype in tumor cells, the underlying mechanisms by which Snail regulates cancer metastatic potential remain elusive.

In A549-S3-KO cells, TGF-β failed to activate RhoA and Rac1 and lentiviral expression of Snail rescued the defect in RhoA activation but not in Rac1 activation (Fig. 4, *B* and *C*). Consistently, knockdown of Snail had no effect on the expression of *ARHGAP24* (Fig. 4*D*), downregulation of which is associated with Rac1 activation in TGF-β–stimulated cells. In addition, Snail failed to rescue the cell motility defect in TGF-β–stimulated A549-S3-KO cells (Fig. 2*D*), whereas it rescued the defect when *ARHGAP2*4 was knocked down (Fig. 4*E*). Therefore, TGF-β–induced cell motility requires Snail-dependent activation of RhoA and independent activation of Rac1 (Fig. 4*F*), both of which are events downstream of Smad3. Snail has recently been reported to inactivate RhoA at intercellular junctions to facilitate clustered cell migration in SAS oral squamous cell carcinoma cells (45). The role of Snail in lung adenocarcinoma cells might differ from that in oral squamous cell carcinoma cells, in which Slug, but not Snail, plays a central role in the induction of EMT-associated cell responses (36, 37, 38).

We further identified Wnt5b as a downstream effector of Snail in cells stimulated with TGF-β. WNT signaling is mediated through at least three different intracellular signaling pathways: the canonical WNT/β-catenin pathway, the WNT/Ca^2+^-dependent pathway, and the WNT/planar cell polarity pathway (27). The noncanonical planar cell polarity pathway is involved in the formation of the actin cytoskeleton through activation of RhoA (46), and Wnt5b is known to be a noncanonical Wnt-ligand (47). In SAS oral squamous carcinoma cells, Wnt5b stimulation alone has been reported to be sufficient for RhoA activation and stress fiber formation (28). However, in the present study, we observed that lentiviral expression of Wnt5b in A549-S3-KO cells was not sufficient to induce cell morphological changes or stress fiber formation; further stimulation with TGF-β was required (Fig. 6, *C* and *D*). The reason for this discrepancy remains to be examined.

Although TGF-β has been previously reported to induce Wnt5b (38, 48), we found that Snail is an upstream regulator of Wnt5b induction by TGF-β in A549 cells. Knockdown of Snail effectively attenuated the upregulation of Wnt5b expression by TGF-β (Fig. 6*A*). Currently, the mechanism by which Snail upregulates Wnt5b expression is insufficiently understood. A luciferase reporter containing a region upstream of exon 1 (∼2.0 kb) in the human *WNT5B* gene failed to respond to TGF-β (M. Motizuki and K. Miyazawa, unpublished observation). However, we noticed that U0126, a MEK inhibitor, attenuated the induction of Wnt5b by TGF-β (Fig. 7*F*). In A549 cells, activation of ERK by TGF-β was sustained as long as 4 h after stimulation; however, when Snail was knocked down, the ERK activation decreased after 1 h post-stimulation (Fig. S5*A*). Intriguingly, Snail-dependent activation of the ERK pathway has been previously reported (49). In addition, the sustained activation of ERK was not observed in A549-S3-KO cells, indicating that it is Smad3-dependent but was rescued by ectopic expression of Snail even though Snail expression itself was not sufficient (Fig. S5*B*). Similarly, Wnt5b induction was not rescued by expression of Snail; additional TGF-β stimulation was required (Fig. S5*C*). Collectively, the results show that TGF-β appears to induce Wnt5b expression *via* the delayed-phase ERK activation with the aid of Snail. The involvement of Wnt ligands in TGF-β–induced invasiveness has been previously reported for breast cancer cells (50), where Wnt7a and Wnt7b were found to be induced by the Smad3–JunB cooperative transcriptional activation. Therefore, Wnt ligands participate in TGF-β–induced cell responses in various ways.

Lentiviral expression of Wnt5b induced stress fiber formation in A549-S3-KO cells in a TGF-β–dependent manner (Fig. 6*D*). The requirement of TGF-β for stress fiber formation implies a signaling pathway other than the Smad3/Snail/Wnt5b pathway. We have previously reported that PI3K activation by TGF-β is independent of Smad3 and that its inhibition by a PI3K inhibitor LY294002 blunts TGF-β–induced Rac1 activation in A549 cells (11). Similarly, LY294002 also inhibits TGF-β–induced RhoA activation (Fig. 3*A*). Phosphatidylinositol-3,4,5-trisphosphate produced by PI3K can recruit proteins with phosphoinositide-binding modules to the plasma membrane (51). Most guanine nucleotide exchange factors for Rho GTPases have such modules; examples include the Pleckstrin homology domains in the Dbl family or the Dock homology region 1 domains in the Dock180 family (52, 53).

As noted above, inhibition of ERK activation by the MEK inhibitor U0126 moderately suppressed the induction of Wnt5b as well as cell morphological changes and stress fiber formation by TGF-β (Fig. 7, *A*, *B* and *F*). However, U0126 effectively suppressed the enhancement of cell motility by TGF-β (Fig. 7*C*). Therefore, the ERK pathway might have function(s) other than induction of Wnt5b in the enhancement of cell motility in A549 cells. Notably, U0126 inhibited the induction of Snail by TGF-β in PANC-1 cells (Fig. S1*E*; see also Ref. (54)), whereas it did not in A549 cells (Fig. 7*F*). Therefore, the roles of MEK–ERK in the Snail pathway appear to vary depending on the cell type.

Inhibition of p38 MAPK with specific inhibitors has also been reported to abrogate the TGF-β–mediated actin reorganization (15, 16, 17). Consistent with these previous reports, we found that a p38 MAPK inhibitor, SB203580, effectively inhibited the TGF-β–induced stress fiber formation in A549 cells (Fig. S3*C*). Although p38 MAPK is known to be activated by the TRAF4/6 pathway (8, 9), we found that p38 MAPK activation at 16 h, but not at 1 h after TGF-β stimulation, is dependent on Smad3 (Fig. S6). Therefore, the activation is likely due to the action of some effector(s) downstream of Smad3. p38 MAPK can regulate actin cytoskeleton dynamics through p38 MAPK/MK2/HSP27 or p38 MAPK/MK2/Lim K/cofilin cascade (55, 56, 57, 58). Nonphosphorylated heat shock protein 27 (HSP27) caps barbed ends of actin filaments and stops the filament elongation (55). However, cofilin is a major actin disassembling factor in the nonphosphorylated state, and its binding to actin is inhibited by phosphorylation catalyzed by members of the Lim kinase or Tes kinase family (56). MAPK-activated protein kinase 2, a downstream kinase of p38 MAPK (57), activates both HSP27 and Lim kinase by phosphorylation (58, 59). p38 MAPK can promote actin polymerization by decapping the barbed end of actin filaments through phosphorylation of HSP27 or by inhibiting the actin-disassembling activity of cofilin through phosphorylation of Lim kinase.

In the present study, we elucidated the role of endogenous Snail in TGF-β–induced EMT, which includes RhoA activation–dependent cell morphological changes, stress fiber formation, and enhanced cell motility *via* induction of Wnt5b in cooperation with non-Smad signaling pathways. Some of the non-Smad signaling pathways (*i.e.*, delayed phase activation of ERK and p38 MAPK) appear to be transmitted indirectly from downstream effectors of Smad3, possibly including Snail and Wnt5b, because these pathways are not activated in A549-S3KO cells after TGF-β stimulation. However, the detailed mechanisms remain to be elucidated. In addition, the mechanism by which Snail induces Wnt5b remains unclear. Nevertheless, we demonstrated that the signaling pathways leading to EMT-associated cell responses branch at Smad3 (Fig. 8). We also found that downregulation of E-cadherin, the best-known hallmark of EMT, is not mediated *via* the Snail pathway in A549 cells stimulated with TGF-β (Fig. 1*A*), although exogenously overexpressed Snail downregulated E-cadherin expression (Fig. 2*A*). Instead, we recently found that ZEB1 plays a critical role in the E-cadherin downregulation by TGF-β (S. Otake and K. Miyazawa, unpublished observation). These findings help reveal the intricate mechanisms by which cancer cells acquire malignant phenotypes by the action of TGF-β.

## Experimental procedures

### Reagents and antibodies

Human recombinant TGF-β1 was obtained from PeproTech and used at 1 ng/ml unless otherwise noted. LY294002 and SP600125 were purchased from Sigma-Aldrich. U0126 was obtained from FUJIFILM Wako Pure Chemical. SB203580 was purchased from Calbiochem. The following antibodies were used: anti–phospho-AKT (9271, Cell Signaling Technology); anti–E-cadherin (610182, BD Bioscience); anti–phospho-ERK (9101, Cell Signaling Technology); anti–phospho-p38 (9211, Cell Signaling Technology); anti–phospho-JNK (9255, Cell Signaling Technology); anti-Rac1 (23A8; Millipore); anti-RhoA (sc-418, Santa Cruz Biotechnology or 2117, Cell Signaling Technology); anti–phospho-Src (2101, Cell Signaling Technology); anti–phospho-Smad2 (134D4, Cell Signaling Technology); anti–phospho-Smad3 (9520, Cell Signaling Technology); anti-Snail (4719, Cell Signaling Technology); anti-Slug (9585, Cell Signaling Technology); anti–α-tubulin (DM1A) (T9026, Sigma-Aldrich); anti-Wnt5a/b (2530, Cell Signaling Technology); and anti–DVL2 (sc-390303, Santa Cruz Biotechnology).

### Cell culture

A549 cells were obtained from the American Type Culture Collection as described previously (60) and authenticated by short tandem repeat analysis. A549-S3-KO was established as previously described (61). Cells were maintained in Dulbecco’s modified Eagle’s medium containing 10% fetal bovine serum, supplemented with 50 units/ml penicillin and 50 μg/ml streptomycin, at 37 °C under a 5% CO_2_ atmosphere.

### Biochemical assays

Cell lysis and immunoblotting were performed as described previously (34). For phosphatase treatment, cell lysates were incubated with alkaline phosphatase (*E. coli* C75) (TAKARA BIO INC.) in the reaction buffer (50 mM Tris-HCl (pH8.8), 1 mM MgCl_2_) for 30 min at 60 °C. Actin stress fiber formation was detected by staining with Rhodamine-conjugated phalloidin (Cytoskeleton), as described in Motizuki *et al.* (62). Chamber migration assay was performed as described in Motizuki *et al.* and Lee *et al.* (11, 33). Quantitative real-time PCR was performed as described in Itoh *et al*. (63). Primer sequences are shown in Table S1.

### Lentivirus production

cDNAs encoding human Snail and Slug were described previously (22). A cDNA encoding human Wnt5b was amplified from A549 cDNA using following primers: forward, 5′-GAGAATTCATGCCCAGCCTGCGCTG-3′; reverse, 5′-GGTCTAGACTATTTACAGAT-GTACTGGTCCACG-3′. A lentiviral vector encoding HA-tagged Snail, HA-tagged Slug, or Flag-tagged Wnt5b was generated by Gateway technology (Invitrogen). Lentivirus particles were produced as previously described (60). Cells infected with Snail or Wnt5b were cloned by limiting dilution in 96-well plates, and multiple clones were used for experiments.

### GTPase activity assay

For Rac1, 2 × 10^5^ cells were seeded on collagen-coated 6-well plates (Cellmatrix Type I-C, Nitta Gelatin), cultured for the indicated time in either the presence or absence of 1 ng/ml TGF-β1, and harvested (11). For RhoA, 1 × 10^6^ cells were cultured for the indicated time in either the presence or absence of 1 ng/ml TGF-β1 on noncoated 6-well plates. Cell lysates were incubated with GST–PAK–CRIB or GST–rhotekin–RBD. Pulled-down complexes were washed and subjected to immunoblotting.

### RNA interference

siRNAs were transfected into cells (2 × 10^5^) using Lipofectamine RNAiMAX transfection reagent (Thermo Fisher Scientific). The stealth RNAi siRNAs against human *ARHGAP24* (#1, 5′-AAGAUAGAGUAUGAGUCCAGGAUAA-3′; #2, 5′-CAGUGGUAAAUUACAACCUCCUCAA-3′); human Slug (#1, 5′-CCGUAUCUCUAUGAGAGUUACUCCA-3′; #2, 5′-GAUGCAUAUUCGGACCCACACAUUA-3′); human Snail (#1, 5′-AGACCCACUCAGAUGUCAAGAAGUA-3′; #2, 5′-CCUGUCAGAUGAGGACAGUGGGAAA-3′); human Wnt5b (#1, 5′-GGCUGUGUAUAAGAUGGCAGACGUA-3′; #2, 5′-GAGAUCGUGGACCAGUACAUCUGUA-3′); and siRNA Negative Control Low GC Duplex were obtained from Invitrogen. The final concentration of siRNA was 10 nM.

### Statistical analysis

A two-sided Student’s *t* test or Dunnett’s multiple comparison test was used to determine the significant differences among the experimental groups. Probability values less than 0.01 were considered significant; ∗*p* < 0.01.

## Data availability

Data are contained within the article and its Supporting information.

## Supporting information

This article contains supporting information (Table S1 and Figs. S1–S6).

## Conflict of interest

The authors declare that they have no conflicts of interest with the content of this article.
