# Supplementary material for: The Snail signaling branch downstream of the TGF-β/Smad3 pathway mediates Rho activation and subsequent stress fiber formation
Source: J Biol Chem. 2023 Dec 21;300(1):105580. doi: 10.1016/j.jbc.2023.105580 (PMC10821601; doi:10.1016/j.jbc.2023.105580)
Supplement: Supporting information [file mmc1.pdf]

Supporting Information for

The Snail signaling branch downstream of the TGF-β/Smad3 pathway mediates Rho activation and subsequent stress fiber formation

Mitsuyoshi Motizuki, Takashi Yokoyama, Masao Saitoh, and Keiji Miyazawa

Supplementary Figures: Fig. S1, S2, S3, S4, S5 and S6

Supplementary Table: Table S1

Fig.S1

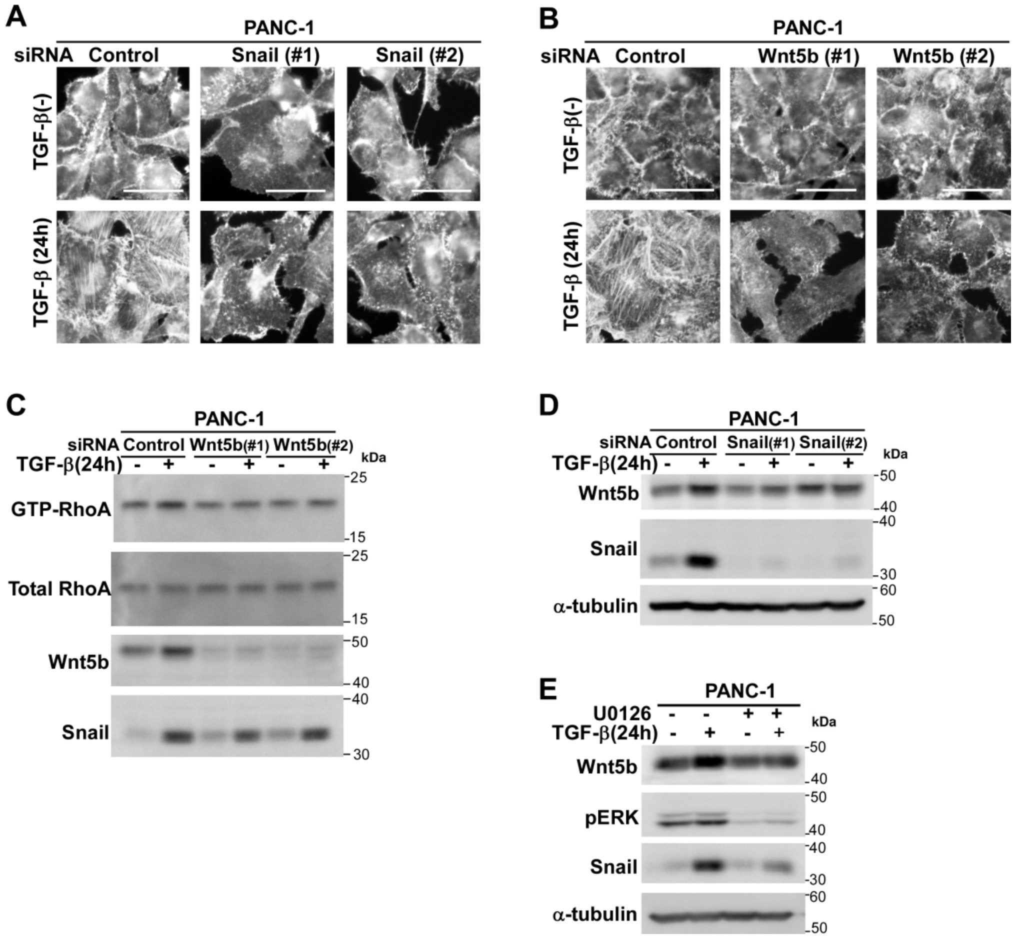

Figure S1. Snail and Wnt5b are crucial for stress fiber formation in PANC-1 cells. *A–D*, PANC-1 cells were transfected with two different siRNAs against Snail (#1 or #2), Wnt5b (#1 or #2), or control siRNA. After 24 h, cells were stimulated with 1 ng/mL of TGF-β1 for 24 h. *A*, the effect of Snail knockdown on TGF-β–induced stress fiber formation. F-actin was stained using Rhodamine phalloidin. *B*, the effect of Wnt5b knockdown on TGF-β–induced stress fiber formation. *C*, TGF-β–induced activation of RhoA was inhibited by knockdown of Wnt5b. RhoA and Wnt5b were detected by immunoblotting. *D*, knockdown of Snail attenuated induction of Wnt5b by TGF-β. Expression of Wnt5b or Snail was examined by immunoblotting; α-tubulin was used as a loading control. *E*, TGF-β–induced expression of Wnt5b as well as Snail was inhibited by U0126. Cells were pretreated with either a MEK inhibitor U0126 (10 μM) or 0.1% dimethyl sulfoxide (DMSO) for 1 h. Expression of Wnt5b, pERK, or Snail was examined by immunoblotting; α-tubulin was used as a loading control. *Scale bars*: 100 μm (*A*, *B*). One representative result from two independent experiments is shown (*A–E*).

Fig.S2

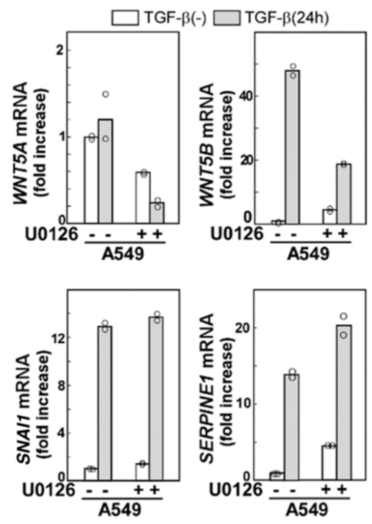

Figure S2. Effects of U0126 on expression of TGF-β target genes. A549 cells were pretreated with either a MEK inhibitor U0126 (10 μM) or 0.1% DMSO for 1 h, stimulated with 1 ng/mL of TGF-β1 for 24 h, harvested, and subjected to quantitative real-time PCR (duplicate determination).

Fig.S3

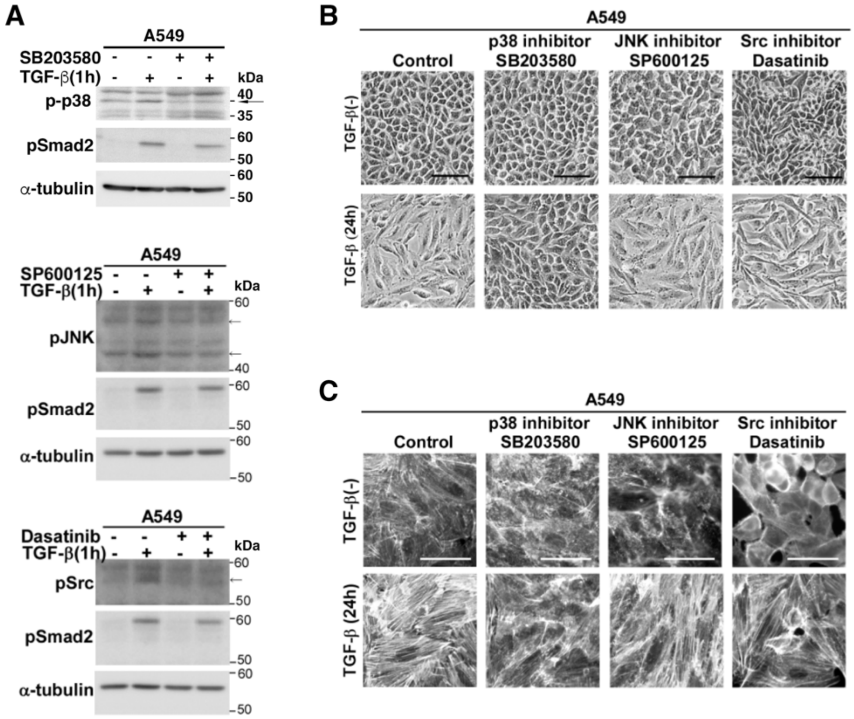

Figure S3. Effects of inhibitors for p38 MAPK, JNK, or Src on TGF-β–induced cell morphological changes and stress fiber formation. *A–C*, A549 cells were treated with SB203580 (10 μM), SP600125 (100 μM), Dasatinib (20 nM), or 0.1% DMSO (vehicle) for 1 h and stimulated with 1 ng/mL of TGF-β1 for the indicated time. *A*, the cells were harvested and assessed using antibodies against phospho-p38, JNK, or Src. *B*, light microscopic images. *C*, formation of actin stress fibers. F-actin was stained using Rhodamine phalloidin. *Scale bars*: 100 μm (*B*, *C*). One representative result from two independent experiments is shown (*A–C*).

Fig.S4

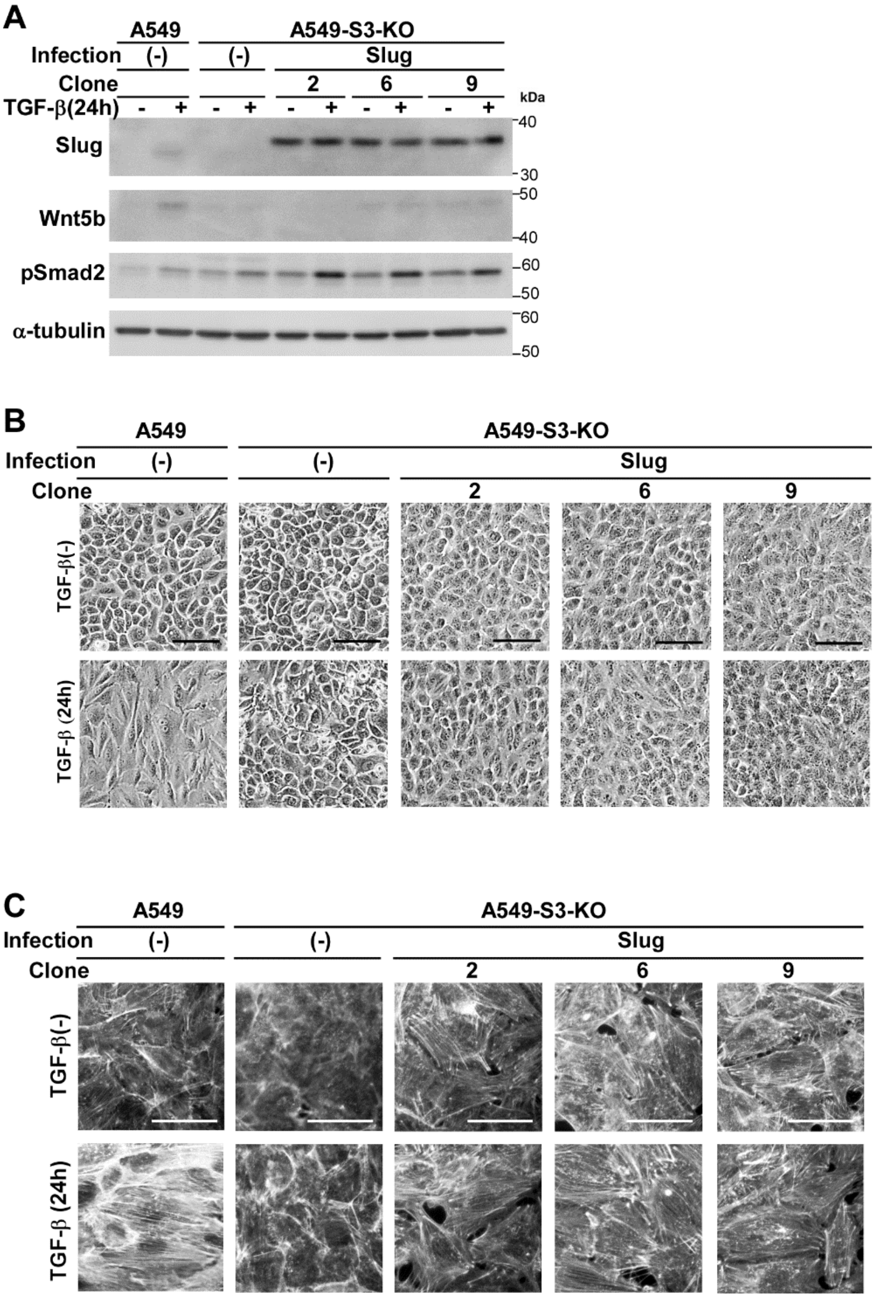

Figure S4. Slug fails to rescue the defect of TGF-β–induced stress fiber formation in *SMAD3*-knockout A549 cells. A549-S3-KO cells were infected with lentivirus carrying HA-tagged Slug encoding cDNA. *A–C*, A549 cells, A549-S3-KO cells, or those expressing Slug were incubated in either the presence or absence of 1 ng/mL of TGF-β1 for 24 h. *A*, expression of Slug, Wnt5b, or phospho-Smad2 was detected by immunoblotting; α-tubulin was used as a loading control. *B*, light microscopic images. *C*, formation of actin stress fibers. F-actin was stained using Rhodamine phalloidin. *Scale bars*: 100 μm (*B*, *C*). One representative result from two independent experiments is shown (*B*, *C*).

Fig.S5

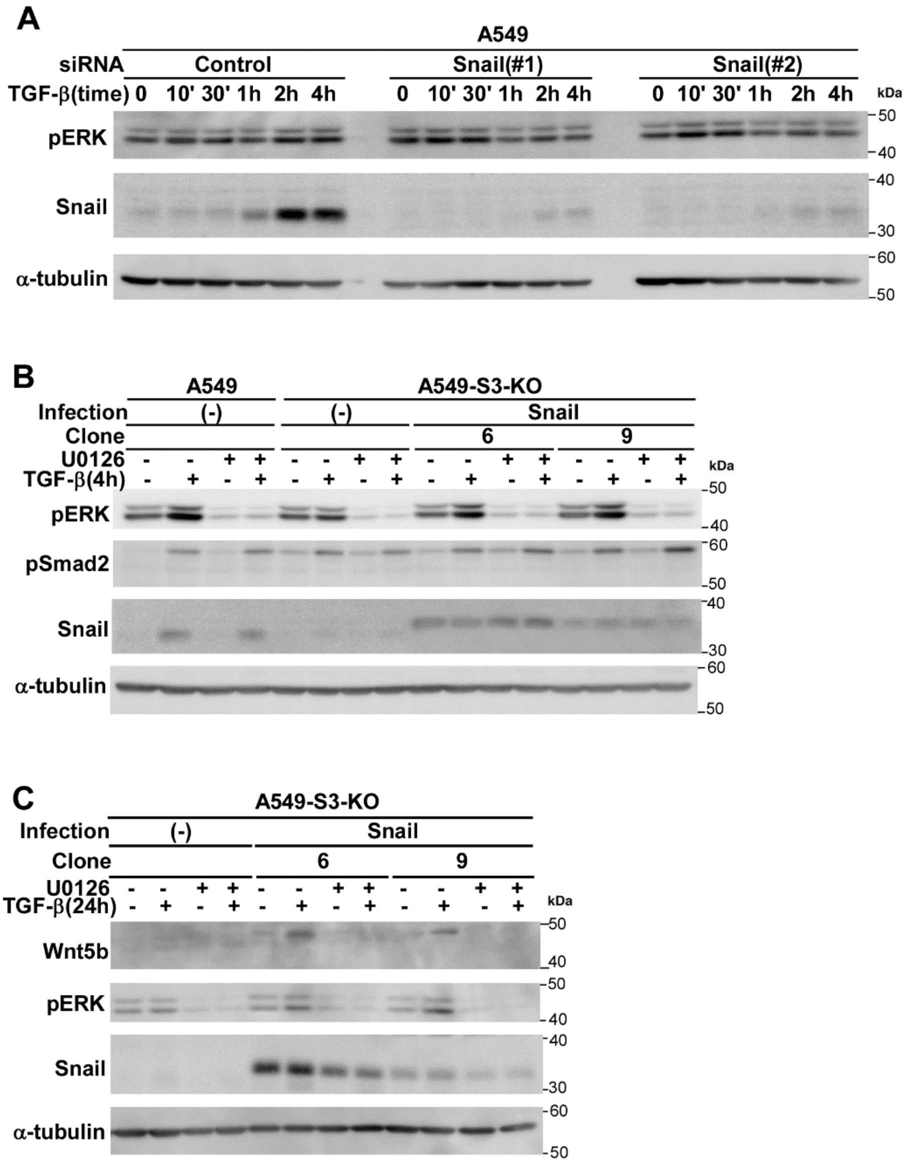

Figure S5. Snail is involved in sustained ERK activation and Wnt5b induction by TGF-β. *A*, effect of Snail knockdown on TGF-β–induced phosphorylation of ERK. Cells were treated with control siRNA (siControl) or siRNA against Snail for 24 h and stimulated with 1 ng/mL of TGF-β1 for the indicated time. *B* and *C*, lentiviral expression of Snail rescued the defect in TGF-β–induced phosphorylation of ERK at 4 h after stimulation (*B*) and upregulation of Wnt5b expression at 24 h after stimulation (*C*) in A549-S3-KO cells. Cells were pretreated with either a MEK inhibitor U0126 (10 μM) or 0.1% DMSO for 1 h. Cell lysates were analyzed by immunoblotting using anti–phospho-ERK, phospho-Smad2, Wnt5b, or Snail antibodies; α-tubulin was used as a loading control. One representative result from two independent experiments is shown (*A*–*C*).

Fig.S6

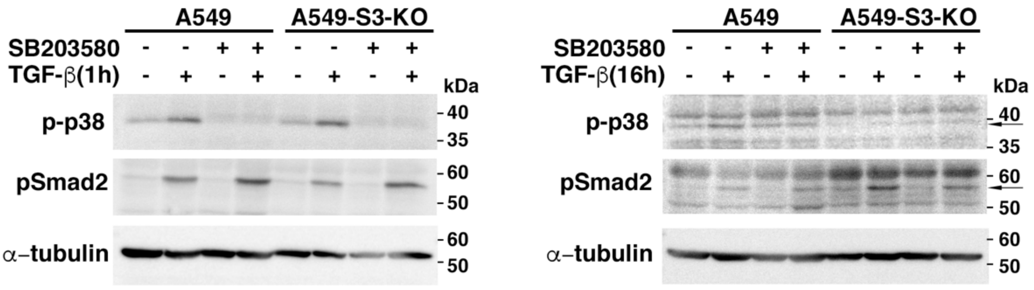

Figure S6. Activation of p38 MAPK at 16 h after TGF-β stimulation depends on Smad3. A549 cells and A549-S3-KO cells were treated with SB203580 (10 μM) for 1 h and stimulated with 1 ng/mL of TGF-β1 for 1 h or 16 h. Phosho-p38 and phosho-Smad2 were detected by immunoblotting. α-Tubulin was used as a loading control. Note that p38 MAPK activation was reduced after 16 h but not 1 h of TGF-β stimulation in *SMAD3*-KO cells.

Table S1. Primers used for quantitative real-time PCR.

	Forward (5'→3')	Reverse (5'→3')
ARHGAP24	TTGTGGCTGTGCTGTTTGTG	GCCTCGCAAAAGCAAAACTG
GAPDH	GAAGGTGAAGGTCGGAGTC	GAAGATGGTGATGGGATTTC
SNAI1	TTCTCACTGCCATGGAATTCC	GCAGAGGACACAGAACCAGAAA
SERPINE1	GGCTGACTTCACGAGTCTTTCA	ATGCGGGCTGAGACTATGACA
WNT5A	CTTCGCCCAGGTTGTAATTGAAGC	CTGCCAAAAACAGAGGTGTTATCC
WNT5B	CTGCCTTTCCAGCGAGAATT	AGGTCAAATGGCCCCCTTT
